# Genomic diversity of antimicrobial resistance in non-typhoidal *Salmonella* in Victoria, Australia

**DOI:** 10.1099/mgen.0.000725

**Published:** 2021-12-15

**Authors:** Cheryll M. Sia, Sarah L. Baines, Mary Valcanis, Darren Y. J. Lee, Anders Gonçalves da Silva, Susan A. Ballard, Marion Easton, Torsten Seemann, Benjamin P. Howden, Danielle J. Ingle, Deborah A. Williamson

**Affiliations:** ^1^​ Department of Microbiology and Immunology, The University of Melbourne at The Peter Doherty Institute for Infection and Immunity, Melbourne, Victoria, Australia; ^2^​ Microbiological Diagnostic Unit Public Health Laboratory, Department of Microbiology & Immunology, The University of Melbourne at The Peter Doherty Institute for Infection and Immunity, Melbourne, Victoria, Australia; ^3^​ Department of Health, Victoria, Australia; ^4^​ National Centre for Epidemiology and Population Health, The Australian National University, Canberra, Australia; ^5^​ Department of Microbiology, Royal Melbourne Hospital, Melbourne, Australia

**Keywords:** *Salmonella*, antimicrobial resistance, whole genome sequencing, public health surveillance

## Abstract

Non-typhoidal *Salmonella* (NTS) is the second most common cause of foodborne bacterial gastroenteritis in Australia with antimicrobial resistance (AMR) increasing in recent years. Whole-genome sequencing (WGS) provides opportunities for *in silico* detection of AMR determinants. The objectives of this study were two-fold: (1) establish the utility of WGS analyses for inferring phenotypic resistance in NTS, and (2) explore clinically relevant genotypic AMR profiles to third generation cephalosporins (3GC) in NTS lineages. The concordance of 2490 NTS isolates with matched WGS and phenotypic susceptibility data against 13 clinically relevant antimicrobials was explored. *In silico* serovar prediction and typing was performed on assembled reads and interrogated for known AMR determinants. The surrounding genomic context, plasmid determinants and co-occurring AMR patterns were further investigated for multidrug resistant serovars harbouring *bla*_CMY-2_, *bla*_CTX-M-55_ or *bla*_CTX-M-65_. Our data demonstrated a high correlation between WGS and phenotypic susceptibility testing. Phenotypic-genotypic concordance was observed between 2440/2490 (98.0 %) isolates, with overall sensitivity and specificity rates >98 % and positive and negative predictive values >97 %. The most common AMR determinants were *bla*_TEM-1_, *sul2*, *tet*(A), *strA-strB* and *floR*. Phenotypic resistance to cefotaxime and azithromycin was low and observed in 6.2 % (151/2486) and 0.9 % (16/1834) of the isolates, respectively. Several multi-drug resistant NTS lineages were resistant to 3GC due to different genetic mechanisms including *bla*_CMY-2_, *bla*_CTX-M-55_ or *bla*_CTX-M-65_. This study shows WGS can enhance existing AMR surveillance in NTS datasets routinely produced in public health laboratories to identify emerging AMR in NTS. These approaches will be critical for developing capacity to detect emerging public health threats such as resistance to 3GC.

## Data Summary

All FASTQ paired-end reads were submitted to National Centre for Biotechnology Information (NCBI) and are available under BioProject PRJNA319593. Supplementary Material is available at Figshare: https://doi.org/10.6084/m9.figshare.16556748.v1 [1] .

**Impact Statement**

In this study, we demonstrated the utilisation of whole genome sequencing in public health in Australia. We evaluated the concordance of inferring phenotypic resistance from genomic data in non-typhoidal *Salmonella* (NTS) from Victoria, Australia collected over the past two decades. We subsequently identified prevalent and emerging antimicrobial resistance (AMR) determinants and profiles associated with NTS serovars. Further, we characterised the genomic context of multidrug resistant NTS isolates harbouring third generation cephalosporin resistance. This study illustrates how genomics has redefined public health surveillance and provides an insight into the population of AMR non-typhoidal *Salmonella* circulating Australia.

## Introduction

Salmonellosis is a major gastrointestinal disease, estimated to cause 93.8 million infections in humans globally per year [2, 3]. The causative agents are serovars of non-typhoidal *Salmonella enterica* subspecies *enterica* (NTS), which have broad animal and environmental reservoirs [4, 5]. Of concern, antimicrobial resistance (AMR) has been increasingly reported in NTS serovars [6, 7]. Due to the global nature of the food supply chain, AMR NTS, including multi-drug resistant (MDR; defined as resistance to ≥3 antimicrobial classes [8]) NTS, has emerged as a global public health threat [5–7, 9].

One of the cornerstones in understanding and potentially interrupting the dissemination of AMR NTS is timely and effective surveillance. For decades, phenotypic antimicrobial susceptibility testing (AST) has been used to monitor AMR in NTS [10–12]. Increasingly, whole genome sequencing (WGS) has been used to complement, or even replace phenotypic susceptibility testing for NTS in public health laboratories [11, 13, 14]. This genomic information enables deeper exploration into the emergence and evolution of distinct serovars, and the genetic context of AMR mechanisms [11, 15]. Previous studies have demonstrated strong concordance between genotypic and phenotypic susceptibility testing for NTS [13, 14, 16], although studies have differed in the phenotypic methods used (e.g. broth microdilution vs agar dilution) and interpretive criteria applied (e.g. Clinical and Laboratory Standards Institute, CLSI vs European Committee on Antimicrobial Susceptibility Testing, EUCAST). As such, results from published studies can be challenging to compare and may not directly extrapolate to other settings.

Australia has one of the highest rates of salmonellosis in the developed world, with recent work highlighting the potential for ‘incursions’ of MDR NTS lineages into Australia [6]. The serious threat posed by MDR NTS, in particular the increasing prevalence of plasmid-mediated AmpC (PMAC) and extended-spectrum beta-lactamase (ESBL) producing NTS strains conferring third-generation cephalosporins (3GCs) resistance [17, 18], has been recognised as one that requires novel strategies for enhanced surveillance and control of emerging threats [19]. To date however, there are limited data assessing the utility of genomics-based surveillance of NTS in Australia, specifically in relation to genotypic AMR surveillance. Accordingly, we sought to (i) assess the ability of genotypic AMR detection to predict phenotypic susceptibility and resistance in the context of a large collection of NTS, and (ii) to identify and investigate emerging 3GCs resistance profiles associated with specific NTS lineages. Our data provides valuable information on the emergence and spread of AMR NTS serovars, and further highlights the utility of genomics for AMR surveillance.

## Methods

### Setting and data sources

In Australia, salmonellosis is a notifiable disease. The Microbiological Diagnostic Unit Public Health Laboratory (MDU PHL) is the bacterial reference laboratory for the State of Victoria in Australia. Phenotypic antimicrobial susceptibility testing was routinely performed on all *Salmonella* isolates, while WGS was only performed when requested until routine WGS commenced in mid-2018. Only isolates that had both phenotypic susceptibilities and WGS data were included in this study. In total, there were 2490 NTS human isolates received at MDU PHL between 1 January 2000 and 31 December 2018 that fit this selection criteria. Details of all isolates in this study are in Table S1 (available in the online version of this article).

### Phenotypic antimicrobial susceptibility testing

Phenotypic antimicrobial susceptibility testing was performed using agar breakpoint dilution. Results were interpreted according to Clinical and Laboratory Standards Institute (CLSI) guidelines [20] (Table S2). Depending on when specific testing was introduced (Table S2), susceptibility of NTS was determined for ampicillin, cefotaxime, meropenem, azithromycin, gentamicin, kanamycin, spectinomycin, streptomycin, chloramphenicol, tetracycline, ciprofloxacin, sulfathiazole and trimethoprim (Table S2). Decreased susceptibility to ciprofloxacin was also determined.

### DNA extraction, whole genome sequencing and genome assembly

DNA extraction was performed using the QIAsymphony DNA Sample Preparation Kit (Qiagen). WGS was performed using Nextera XT as per manufacturer’s instruction. Paired-end reads of 150 bp were generated using the Illumina NextSeq 500/550 platform (Illumina, San Diego, CA), while 300 bp paired-end reads were generated using the Illumina MiSeq platform (Illumina, San Diego, CA). Reads were trimmed and quality checked using Trimmomatic [21], before they were *de novo* assembled using Shovill (used v1.0.1, v1.0.3, v1.0.4 and v1.0.9) (https://github.com/tseemann/shovill), which subsamples the reads to a minimum of 18× coverage and then assembles using SPAdes (used v3.12.0, v3.13.0 and v3.13.1) [22]. The version of each tool was dependent on the pipeline used at the time WGS was performed. All assembled reads were subjected to the following quality checks: a minimum Phred quality score >30, read coverage >40× and an estimated genome size between 4.2–5.2 Mbp before they were deposited to the MDU PHL *Salmonella* database. FASTQ paired-end reads are available at BioProject PRJNA319593.

### *In silico* detection of AMR determinants, multi-locus sequence type and serovar

Genome assemblies were screened for known AMR determinants in the National Center for Biotechnology Information (NCBI) Bacterial Antimicrobial Resistance Reference Gene Database 2019-09-07 using ABRicate v0.9.8 (https://github.com/tseemann/abricate) with a minimum nucleotide identity (ID) of 95 % and minimum coverage (COV) of 100%, which was internally validated for this study using different threshold combinations as the optimal threshold for identifying AMR determinants for this pipeline. Known point mutations associated with AMR were detected using Antimicrobial Resistance Identification by Assembly (ARIBA) v2.13.3 [23] against the Comprehensive Antibiotic Resistance Database (CARD) v3.0.1 [24] at a lower identity threshold of 90 % ID and 100 % COV to ensure all possible point mutations were identified. These specific point mutations were in the quinolone resistance determining region (QRDR) of the DNA gyrase (*gyrA* and *gyrB*) and topoisomerase (*parC* and *parE*) subunits [16, 25]. The output from ABRicate was then tabularised and summarised using Pandoo (https://github.com/schultzm/pandoo), while ARIBA utilised its in-built *summary* tool. Only AMR determinants with a known phenotype were analysed in this study.

Genome assemblies were screened to infer multi-locus sequence type (ST) using *mlst* v2.16.1 (https://github.com/tseemann/mlst) using default parameters with the *S. enterica* MLST scheme [26]. Serovars for all NTS genomes were determined using *Salmonella in silico* Typing Resource (SISTR) v1.1.1 [27].

### Correlation of phenotypic and genotypic AMR

Concordance between antimicrobial susceptibility phenotype and the AMR genotype was assessed by comparing susceptibility profiles for each antimicrobial with the presence of known AMR determinants. Very major errors (VME, phenotypically resistant but genotypically susceptible) and major errors (ME, phenotypically susceptible but genotypically resistant) were distinguished [28]. Resulting graphs and plots were visualised using *ggplot2* v3.2.1 [29]. The frequency of the detected AMR genes for each NTS lineage, defined by inferred serovar and ST, was also calculated (https://github.com/Siacm/Phenotypic-genotypic-correlation). The sensitivity, specificity, positive predictive value (PPV) and negative predictive value (NPV) of predicting genotypic resistance was determined, with a 95 % confidence interval, for each antimicrobial. Repeat susceptibility testing was performed on all discordant isolates, with their corresponding WGS reads re-screened for AMR determinants using ARIBA at 95 % ID and 100 % COV. This was done to establish whether a combined mapping/alignment approach that uses paired end reads as input would improve discrepancies.

### Phylogenomic analysis of core genome

To show the distribution of 3GC resistance determinants amongst the most resistant NTS isolates in this study, a maximum likelihood (ML) phylogenetic tree of all MDR NTS isolates was inferred to provide a basic population structure. NTS isolates that were defined as MDR were mapped to a publicly available reference genome, *S*. Typhimurium ST19 LT2 strain (NCBI accession AE006468.2) using Snippy v4.4.6 (https://github.com/tseemann/snippy) with a minimum read coverage of 10× at variant sites and a proportion of variant evidence of 0.90, which is part of the Nullarbor pipeline (v2.0.20191007). The resulting core genome SNP alignment was then used to infer a phylogenetic tree with IQ-TREE v1.6 [30] with ultrafast bootstrapping with 1000 replicates [31] using the GTR+G4 substitution model. The resulting ML tree was then visualised using *ggtree* R package [32].

### Genome annotation, plasmid detection and analysis of surrounding genomic region of 3GCs genes

The genetic context of isolates harbouring either *bla*_CMY-2_, *bla*_CTX-M-55_ or *bla*_CTX-M-65_, the three most common 3GC resistance determinants was further explored. Prokka v1.14.6 [33] was used to annotate and identify assembled contigs harbouring *bla*_CMY-2_, *bla*_CTX-M-55_ or *bla*_CTX-M-65_, while Artemis v18.1.0 [34] and blastp was used to visualise and identify flanking coding sequences, respectively. Annotations of insertion sequences (IS) were checked against the ISfinder database 2021-01-15 [35]. Figure annotations were created using DnaFeaturesViewer v3.0.3 python script (https://edinburgh-genome-foundry.github.io/DnaFeaturesViewer/). Plasmid replicons were detected in the assemblies using ABRicate v0.9.8 in conjunction with the PlasmidFinder [36] database with a 80 % ID and 95 % COV threshold. These cut-offs were in line with the author’s recommendations [36] but were adjusted to a higher coverage threshold to determine the best matching replicon. PlasmidSPAdes v3.15.0 [37] was used with default parameters to *de novo* assemble putative plasmids in all isolates harbouring *bla*_CMY-2_, *bla*_CTX-M-55_ or *bla*_CTX-M-65_. The assembly graphs of the reconstructed plasmids were viewed using Bandage [38] and were used as input for NCBI blastn to search for a reference plasmid of the same plasmid replicon type with a similar backbone. Mauve v2.4.0 [39] was then used to reorder the contigs of each isolate harbouring *bla*_CMY-2_, *bla*_CTX-M-55_ or *bla*_CTX-M-65_ to the reference plasmid and visualised using Blast Ring Image Generator (BRIG) v0.95 [40]. Plasmid sequences that aligned to >90 % coverage of the reference plasmid backbone were identified as the putative plasmid harbouring *bla*_CMY-2_, *bla*_CTX-M-55_ or *bla*_CTX-M-65_.

### Co-occurrence networks of AMR genes

In order to investigate the co-occurrence of AMR determinants, pairwise co-occurrence matrices were constructed for the most common 3GC resistance encoding plasmid types. Co-occurrence networks were visualised in R using *igraph* v1.2.4.1 [41]. The nodes represent genes, and the frequency of the co-occurrence of genes exceeding a given threshold are represented by the connecting branches (edges). Thresholds were analysed using a standard deviation (SD) of AMR gene content for each NTS serovar using an approach previously described [42].

## Results

### Phenotypic AMR profiles

**Fig. 1.**
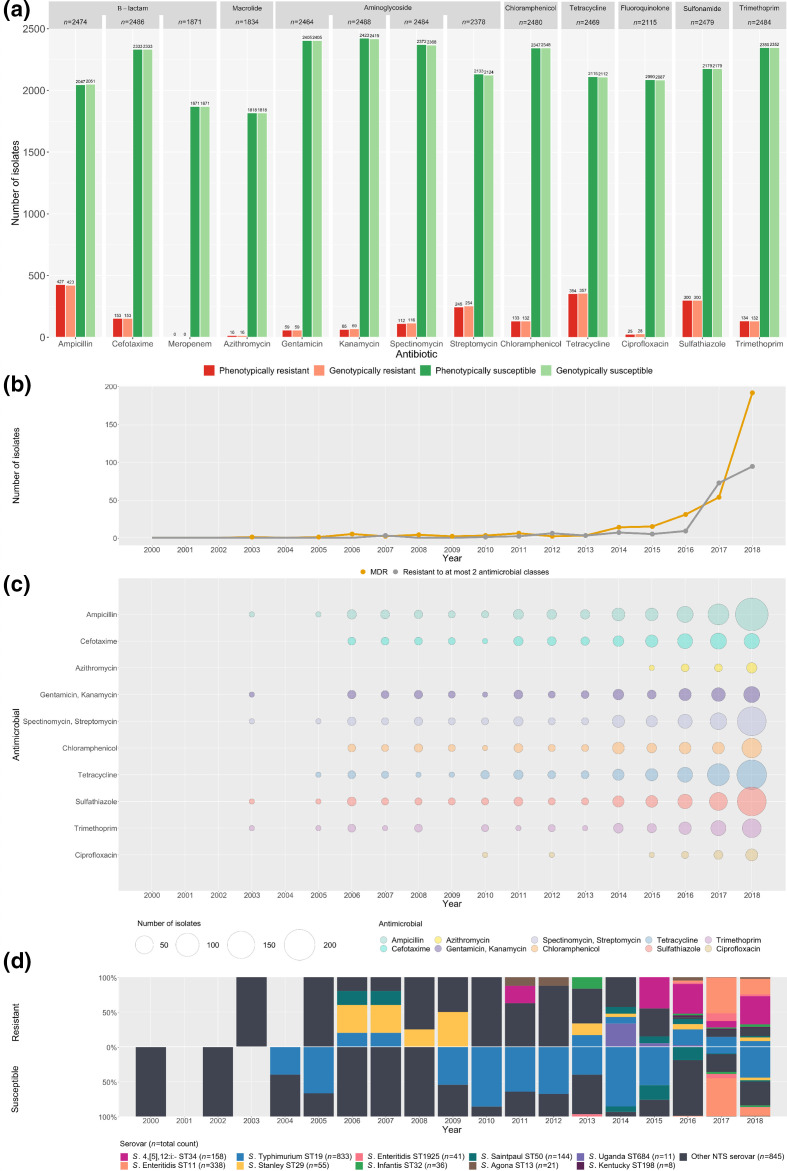
Overall AMR profiles from genomic and phenotypic data. (**a**) Bar plot representing all phenotypic susceptibilities and concordant genotypic susceptibilities for each antimicrobial (*x*-axis). The height of each bar corresponds to the number of isolates (*y*-axis) with its respective count labelled on top of each bar. Each drug has been categorised according to its antimicrobial class with the overall number of isolates (***n***) tested for that drug. (**b**) Number and distribution of phenotypic isolates demonstrating resistance to at most two antimicrobial classes and multidrug resistance over a timespan of 18 years. (**c**) Diverse pattern of phenotypic resistance to particular antimicrobials over a timespan of 18 years. (**d**) Phenotypic susceptibility profiles of top ten resistant serovars over a time span of 18 years.

In total, 540/2490 (21.7 %) isolates were phenotypically resistant to at least one antimicrobial (Fig. 1, Table S2). Resistance was highest for ampicillin (427/2474; 17.3%), followed by tetracycline (354/2469; 14.3%), sulfathiazole (300/2479; 12.1%) and streptomycin (245/2378; 10.3%). Resistance to cefotaxime, was low (153/2486; 6.2%), as was resistance to azithromycin (16/1834, 0.9%) and ciprofloxacin (25/2113; 1.2%). Intermediate susceptibility to ciprofloxacin was detected in a further 375/2490 isolates (15.1%). A total of 336 isolates (13.5%) were phenotypically considered to be MDR defined as resistance to three or more antimicrobial classes [8] (Table S1). No isolates were phenotypically resistant to meropenem.

### High concordance between AMR phenotypes and genotypes

A total of 2440/2490 (98.0 %) isolates had concordant phenotypic and genotypic AMR profiles, based on 30 506 possible isolate and antimicrobial combinations (Fig. 1, Table S2). The overall sensitivity (AMR phenotype and genotype both resistant) was 98.7 % and had a PPV of 97.94%, while the specificity (AMR phenotype and genotype both susceptible) was 99.9 % and an NPV of 99.91 % (Fig. 1, Table S2). Azithromycin had the lowest sensitivity and PPV (both 87.5%) with previously described AMR determinants detected in 14/16 phenotypically resistant isolates. Overall, 50 isolates had discordant results, comprised of 68 phenotype-genotype mismatches. Of these 50 isolates, 16 (32.0 %) isolates were classified as having VME, 32 (64.0 %) had ME and two (4.0%) isolates were found to exhibit VME for one antimicrobial and ME for another antimicrobial. There were seven isolates that had VME to at least two antimicrobials, with the remaining 11 VME isolates discordant for one antimicrobial (Table S3). Of the 34 isolates with ME, eight of these had ME to two different antimicrobials. The antimicrobial with the most ME (24.0%, *n*=10) was streptomycin with the detection of *aadA* genes (*n*=9) and a susceptible phenotype. A *bla*_TEM_ gene with 99.8 % identity to *bla*_TEM-12_ was responsible for both cefotaxime ME discrepancies (Table S3). Both isolates had a non-synonymous mutation at amino acid position 162:S-R relative to *bla*_TEM-12_ (Genbank accession NG_050163.1) suggesting the possibility of a novel *bla*_TEM_ allele that does not confer resistance to 3GCs. Repeat phenotypic susceptibility testing and WGS analysis confirmed these discrepancies. Overall, the specificity of AMR prediction exceeded 99.5 % in all antimicrobials tested.

### Distribution of genomic determinants of antimicrobial resistance

**Fig. 2.**
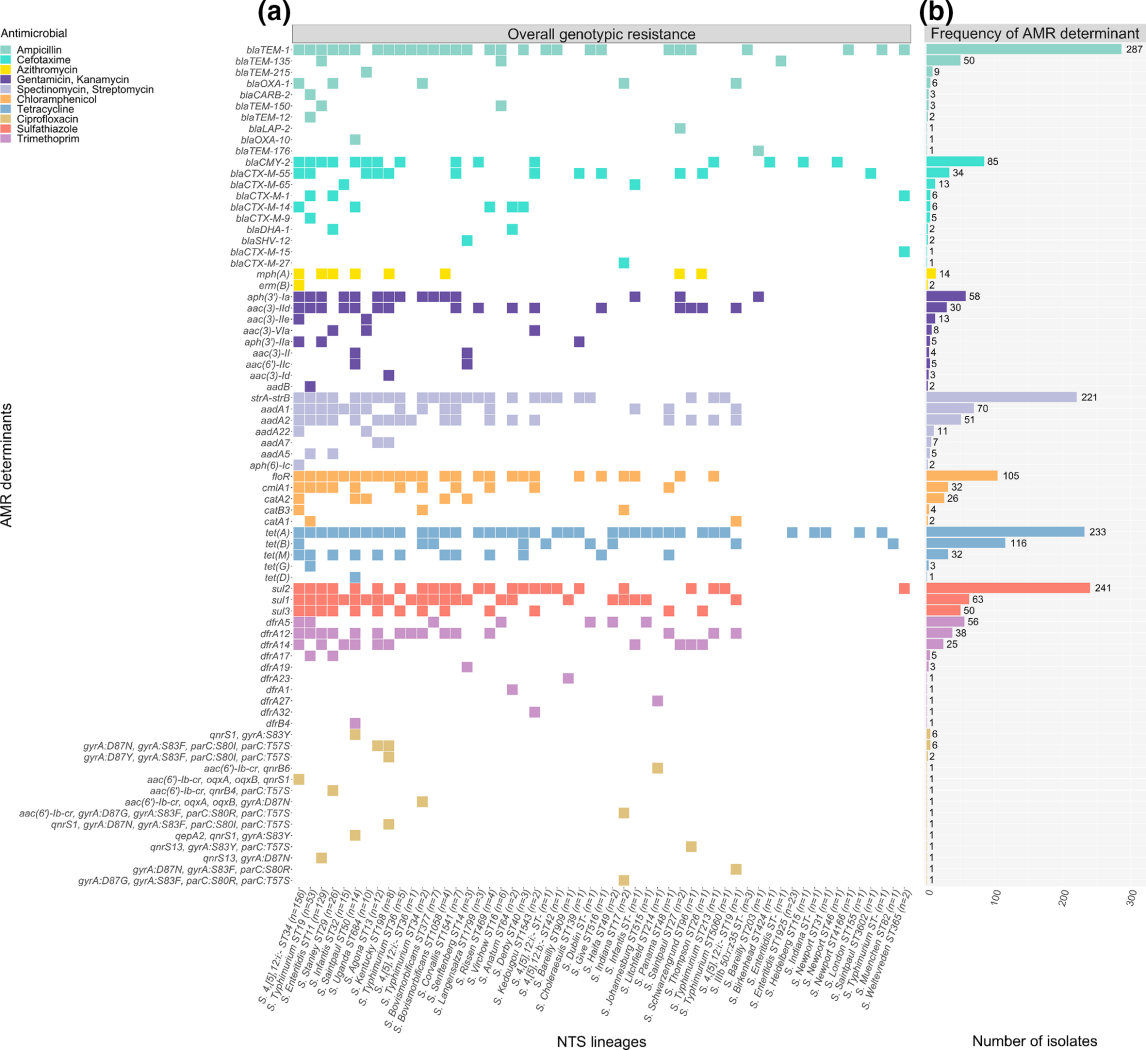
Distribution of AMR determinants by antimicrobial class. (**a**) Heatmap of combined overall genotypic AMR profiles observed for each NTS strain. This is the broad AMR profile of each lineage and not just a single resistant isolate. The total number of isolates for each NTS strain is labelled in closed parenthesis (*x*-axis). The AMR genes (*y*-axis) are coloured according to their antimicrobial target. (**b**) Bar plot demonstrating the frequency of AMR determinants arranged in decreasing order according to each antimicrobial target. The values at the ends of each bar plot refer to the number of isolates harbouring that AMR determinant. AMR genes that conferred resistance to multiple antimicrobials were grouped into one category i.e. spectinomycin and streptomycin. (AMR, antimicrobial resistance; NTS, non-typhoidal *Salmonella *).

In total, 94 serovars were detected using *in silico* serovar prediction (Table S1). Amongst these 94 serovars, 77 different genomic AMR determinants were identified. This included two efflux pump genes, eight unique point mutations in QRDRs and 67 acquired resistance genes (Fig. 2, Table S4). The diversity of AMR genotypes was further reflected in 166 different combinations of AMR determinants within the 2490 NTS isolates. The most common genes mediating AMR were *bla*_TEM_ (*bla*_TEM-1_ (*n*=287) and *bla*_TEM-135_ (*n*=50); ampicillin resistance), *strAB* (*n*=222; streptomycin resistance), *sul* (*sul1* (*n*=63) and *sul2* (*n*=241); sulfathiazole resistance)*, tet* (*tet*(A) (*n*=233) *and tet*(B) (*n*=116); tetracycline resistance) and *floR* (*n*=105; chloramphenicol resistance) (Fig. 2, Table S4). Ciprofloxacin resistance was most frequently mediated by point mutations in the QRDR region of the *gyrA* and *parC* genes (*n*=13), although in some isolates (*n*=9) the resistance profile was due to a single point mutation and an acquired *qnr* gene. Reduced susceptibility to ciprofloxacin, determined by either one or two point mutations in QRDRs or the presence of *qnrs1* (Table S1), was uncommon in the NTS. These profiles were observed in 369/2490 (14.8 %) from 36 different serovar ST lineages. However, nearly two thirds of these isolates were *S*. Enteritidis ST11 (*n*=238) with the single QRDR point mutation *gyrA*_D87Y_ detected in 126 isolates and *gyrA*_D87N_ detected in 94 isolates. The next most common serovar ST was I 4,[5],12:i:- ST34 where the *qnrs1* was detected in 22/26 isolates with inferred reduced susceptibility. Other intermediate combinations included a single plasmid mediated quinolone resistance (PMQR) gene with or without *parC*[57:T-S] (Table S5). The *mph*(A) gene was detected in all fourteen concordant isolates that were resistant to azithromycin. Resistance to 3GCs was mediated by *bla*_CMY-2_ (*n*=81), variants of *bla*_CTX-M_ (*n*=62), or both (*n*=4) (Table S5).

Several lineages were defined by AMR determinants unique to that lineage. For example, *S*. Senftenberg ST14 was the only lineage where *bla*_SHV-12_ and *dfrA19* were detected. Isolates from *S*. Typhimurium ST19 harboured class A β-lactamase genes *bla*_TEM-12_ and *bla*_CTX-M-9_ in addition to chloramphenicol *O*-acetyl transferase *catA1*, *bla*_CARB-2_ and *tet*(G). The latter two genes have been associated with *Salmonella* genomic island I [43–45]. Nearly all the *bla*_TEM-135_ genes (*n*=48/50) in the NTS dataset were identified in *S*. Enteritidis ST11. Moreover, chromosomal QRDR point mutations in the *gyrA* subunit were primarily observed in *S*. Enteritidis ST11 (*n*=235), while the QRDR point mutation profile *gyrA*[83:S-F], *gyrA*[87:D-Y], *parC*[80:S-I], *parC*[57:T-S] was unique to *S*. Kentucky ST198. Other lineages were notably characterised by a lack of any AMR determinants, such as *S*. Dublin ST10 and *S*. Newport ST31.

### Emergence of third generation cephalosporin resistance in multidrug resistant serovars

Of the concordant isolates that conferred 3GC cefotaxime resistance, 84.8 % (*n*=128/151) were identified as MDR. The 3GC resistant isolates primarily carried *bla*_CMY-2_ (*n*=68), *bla*_CTX-M-55_ (*n*=32) or *bla*_CTX-M-65_ (*n*=13); accordingly, we specifically investigated AMR profiles in MDR lineages harbouring any of these 3GC resistance determinants. The emergence of these 3GC resistance genes was frequently associated with the presence of distinct plasmid replicons (Table S6).

**Fig. 3.**
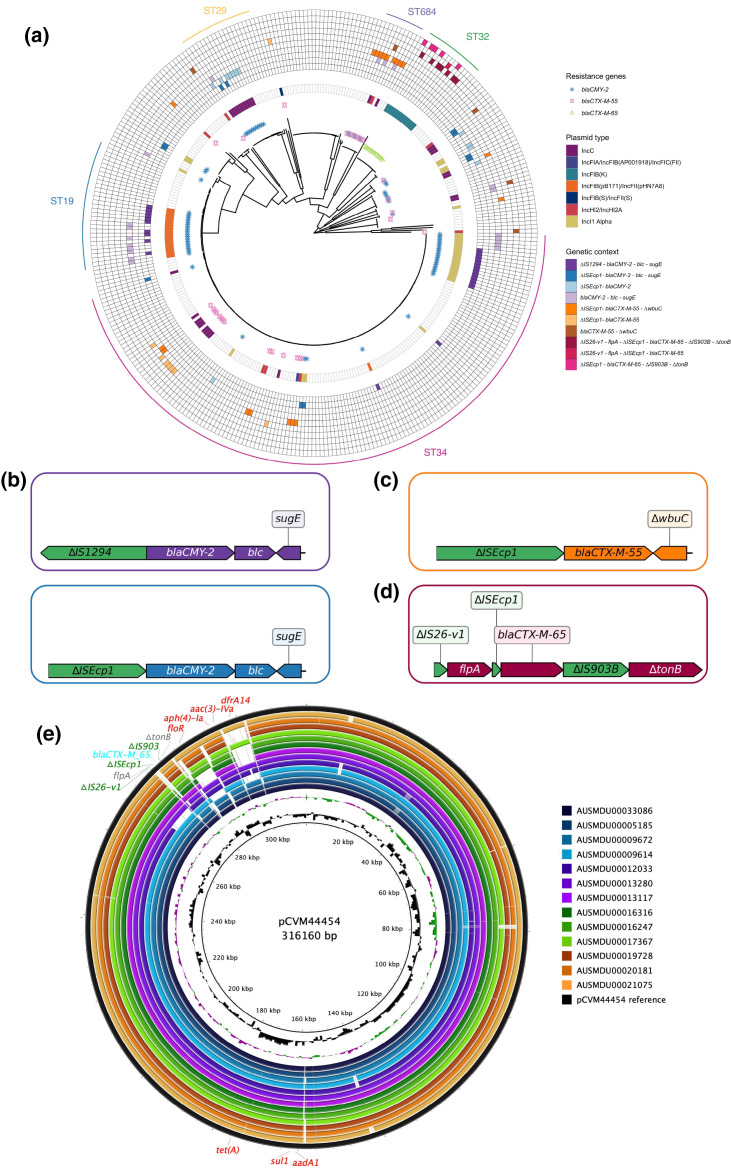
Inferred population structure and genomic profiles of *bla_CMY-2_*, *bla_CTX-M-55_* and *bla_CTX-M-65_* within 334 MDR NTS from Australia. (**a**) Phylogenetic tree of 334 MDR isolates with the distribution of third-generation cephalosporins (3GCs) resistance determinants bla_CMY-2_, bla_CTX-M-55_ and bla_CTX-M-65_. The ST34 clade is comprised of both monophasic and biphasic *S*. Typhimurium ST34 strains. Individual antimicrobial resistance genes form the inner coloured shapes of the tree. The inner ring refers to the plasmid type detected. The outer rings refer to the different genetic environments observed surrounding bla_CMY-2_, bla_CTX-M-55_ and bla_CTX-M-65_. (**b**) Genetic context of the two main transposition units seen in bla_CMY-2_-positive MDR isolates. (**c**) Genetic context of the main transposition unit seen in bla_CTX-M-55_-positive MDR isolates. (**d**) Genetic context of the main transposition unit seen in bla_CTX-M-65_-positive MDR isolates. (**e**) BRIG plot demonstrating an IncFIB(K) plasmid harboured by *S*. Infantis isolates from this study aligned to the U.S reference plasmid, pCVM44454. (ST, sequence type; MDR, multidrug resistant).

The surrounding genomic region and plasmid profiles differed between the serovars in which the *bla*_CMY-2_ gene was detected. In MDR *S*. Stanley ST29, the IncC plasmid replicon was detected in a cluster of isolates with the *bla*_CMY-2_ gene flanked by a truncated (∆) IS*Ecp1* and genes *blc-sugE* (Fig. 3a, b). In contrast, the genetic environment of *bla*_CMY-2_ in a cluster of MDR *S*. Typhimurium ST19 and *S*. 4,[5],12:i:- ST34 isolates was found to be flanked by ∆IS*1294* and *blc-sugE* (Fig. 3a, b). The plasmid replicons also differed, with IncFIB(pB171)/IncFII(pHN7A8) detected in *S*. Typhimurium ST19 and IncI1 Alpha in *S*. 4,[5],12:i:- ST34 (Fig. 3a, b, Table S6). None of the plasmid sequences in these isolates aligned to known plasmids (IncC: pYU07-18 (CP035548.1), pSE12-01738-2 (CP027679.1), p28322-2 (CP051360.1), p15C38-2 (LC501585.1), pST56-1 (CP050740.1); IncFIB/IncFII: pJJ1987_1 (CP013836.1), pSC138 (NC_006856.1), p33673 (CP012683.1), pHH12_P2 (CP058915.1), IncI: pS10584 (KX058576.1)).

A cluster of eight MDR *S*. 4,[5],12:i:- ST34 isolates with an IncC replicon harboured *bla*_CTX-M-55_, and primarily co-occurred with *floR*, *tet*(A) and *qnrS1* (Fig. 3a, Table S6, Fig. S1). Conversely, 50 % (*n*=5/10) of MDR *S*. Uganda ST684 isolates encoding *bla*_CTX-M-55_ did not co-occur with *qnrS1* but instead *bla*_CTX-M-55_ was co-located on the same contig as *bla*_TEM-215_. ∆IS*Ecp1* was found upstream of *bla*_CTX-M-55_ in all but five *bla*_CTX-M-55_-positive MDR isolates (Fig. 3c). The presence of the genetic structure ∆IS*Ecp1-bla*_CTX-M-55_ in isolates without a plasmid type identified by PlasmidFinder suggests that *bla*_CTX-M-55_ could have integrated in the chromosome. Similarly, the plasmid sequences were not associated with known plasmids (IncC: pST101-1 (CP050732.1), p16E080 (NZ_MN647788.1), pVb0499 (NZ_MF627445.1), IncFIB/IncFII: pN16EC0879-1 (CP043745.1), FDAARGOS_446 (CP023947.1), IncHI2/IncHI2A: pMTY18780-1 (AP023198.1), pPJM1 (MN539017.1)).

The 13 MDR *S*. Infantis isolates were shown to carry a MDR IncFIB(K) plasmid which included the *bla*_CTX-M-65_ gene (Fig. 3e). These plasmids were highly similar to pCVM44454 (Genbank accession CP016413.1), a pESI (plasmid for emerging *S*. Infantis, NZ_CP047882.1)-like MDR plasmid detected from a human isolate in the U.S [46] (Fig. 3a, e). The genomic context surrounding the *bla*_CTX-M-65_ gene was consistent in all *S*. Infantis isolates and was identified as ∆IS*26*-v1-*flpA*-∆IS*Ecp1- bla*_CTX-M-65_-∆IS*903B-∆tonB* (Fig. 3d, e). The co-occurrence AMR gene pattern that defined *S*. Infantis was *bla*_CTX-M-65_, *aadA1*, *sul1* and *tet*(A) and the IncFIB(K) replicon (Fig. S1c).

## Discussion

Here, we demonstrate the utility of WGS data for inferring AMR profiles from a collection of 2490 NTS. We observed high sensitivity (98.7%), specificity (99.9%), PPV (97.9%) and NPV (99.9%) rates for concordance between genomic and phenotypic AMR profiles. These findings are consistent with studies exploring AMR prediction from WGS in foodborne enteric pathogens [13, 14, 47–52]. Notably, azithromycin had the lowest sensitivity and PPV rates in our dataset, similar to findings in other studies [53–55]. This may in part be due to the lack of specific MIC thresholds for this drug in NTS as CLSI only specifies interpretive criteria for typhoidal *Salmonella* serovars [20, 56]. Some AMR determinants, such as *tet*(M) and *aadA* variants, have also shown to exhibit low levels of resistance [57, 58]. Further, novel mechanisms such as upregulation of efflux pumps have been putatively identified as mediating resistance to azithromycin, although additional experimental work is required to demonstrate phenotypic resistance [56, 59, 60]. The parameters used in this study adhered to recommended cut-off thresholds, but were limited to the detection of known AMR mechanisms present in public AMR reference databases [56]. Novel AMR mechanisms are commonly identified from discordant phenotypic susceptibilities [13, 61], which would be missed when relying on either phenotypic or genotypic susceptibilities alone. As such, regular phenotypic and genotypic characterisation of AST amongst NTS should be performed in public health laboratories to ensure novel mechanisms of resistance are still detected.

In addition to detection of AMR mechanisms, genomic-based surveillance can be used for the early identification of ‘incursions’ and spread of MDR NTS. For example, we identified several MDR lineages known to disseminate internationally, such as the epidemic ciprofloxacin-resistant *S*. Kentucky ST198 lineage that originated from Africa and has recently been classified as a high-risk pathogen by the World Health Organization [19, 62, 63]. NTS serovars *S*. Dublin and *S*. Newport, which have exhibited increasing rates of MDR overseas [64–66], had low AMR rates in our study. This highlights the need to identify the emergence of MDR lineages in Australia and continuously monitor AMR within these lineages. The greater resolution afforded from genomic-based AMR profiles is critical for future surveillance of ‘one health’ pathogens, such as MDR NTS, for which multiple animal reservoirs exist and which are intricately linked with the global food chain [67].

Although 3GC genes were relatively rare in our dataset, we identified variants of *bla*_CMY_ and different *bla*_CTX-M_ genes known to be associated with human and animals NTS infections in other countries [67–71]. For example, the IS*Ecp1* transposition unit (TPU) associated with *bla*_CMY-2_ and *blc-sugE* in IncC plasmids have been reported in chicken, cow and human NTS isolates from North America and Asia [72], while *bla*_CTX-M-55_ (in conjunction with an IncC replicon) in the *S*. 4,[5],12:i: ST34 lineage has been associated with retail meats, particularly pork products, from several Asian countries [68, 71, 73]. The MDR *S*. Infantis ST32, characterised by the *bla*_CTX-M-65_ gene and IncFIB(K) plasmid replicons, has been detected in both European and North America in recent years from both humans and animals [46, 74, 75]. Further, the IS*1294-bla*_CMY-2_ TPU have been identified on IncI and IncF plasmids worldwide from different members of Enterobacteriaceae [67, 76–79]. The predominance of *bla*_CTX-M-55_ in comparison to *bla*_CTX-M-9_ and *bla*_CTX-M-14_, which were the leading *bla*_CTX-M_ variants found previously in Australian livestock, indicate the emergence of *bla*_CTX-M-55_ from imported meat or overseas travel [68–71, 73]. The increase of MDR lineages that confer resistance to clinically relevant antimicrobials, as seen in *bla*_CTX-M-55_ isolates co-occurring with *qnrS1*, have been observed in NTS isolates from Asia [68, 73] and poses a serious public health threat. Incursions of 3GCs-producing MDR NTS lineages could result in outbreaks where antimicrobial treatment options will be limited. Applying AMR surveillance to food pathogen genomic databases, such as GenomeTrakr [80], could provide the ability for early genomic detection of emerging 3GC resistant NTS lineages in Australia and offer a significant opportunity to rapidly identify transmission events and limit potential outbreaks in the future.

A key strength of this study is the temporal span and scale of the NTS dataset. The two-decade time span shows the application of genomic surveillance in the detection of emergence of AMR NTS in Australia. Similar to other studies, a limitation of our study is that we only assessed known point mutations. Recent studies have shown that point mutations in regulatory genes associated with efflux pumps lead to an altered porin expression [59, 60]. However, future work is required to investigate efflux pumps in NTS before these could be included in genomic AMR surveillance. As a result, discordant profiles may be a result of novel AMR mechanisms or novel genes that have not yet been included in publicly available databases. Further, the lack of breakpoint interpretation in the CLSI guidelines for NTS in some antimicrobials, such as azithromycin, have also contributed to discrepant combinations [20, 81]. Future research in characterising novel AMR mechanisms in NTS both from genomic analyses and phenotypic experiments will help to further refine inferring AMR profiles from WGS data.

Our study also demonstrates the different transposons and plasmids found in association with *bla*_CMY-2_, *bla*_CTX-M-55_ and *bla*_CTX-M-65_ in several MDR NTS lineages, and highlights the major role that mobile genetic elements such as transposons, class 1 integrons and plasmid resistance islands, contribute to the emergence of MDR in NTS, both in Australia and globally [13, 14, 82–90]. Integrating AMR and plasmid profiles with the ST and serovar, provides a means of early detection for emerging MDR profiles that could represent serious public health threats. For example, in our data we identified the international *S*. Infantis pESI-like MDR plasmid [46]; this is the first time this has been reported in Australian NTS isolates. Mobile elements may also integrate into the chromosome as has been previously reported for *bla*_TEM-1_, *strA-strB*, *sul2* genes mobilised on Tn*6029* in *S*. 4,[5],12:i:- [91]. This has resulted in the characteristic ASSuT AMR profile that has historically acted as an epidemiological marker for this lineage [91, 92]. Further work with long read sequencing would resolve the limitations in this study involved in analysing regions flanked by IS, such as *bla*_CTX-M-55_ [93], and provide a more detailed insight into the integration and transmission of these mobile regions.

In summary, we demonstrate a high concordance (99.8%) between genotypic and phenotypic AMR profiles in NTS and highlight how WGS data can be utilised to identify emerging AMR NTS in Australia. Our data further highlight the utility of inferring AMR profiles for NTS from WGS data routinely produced in public health laboratories. Our data provide a framework for the ongoing genomic surveillance of AMR in NTS in our setting; and may potentially act as an early warning system for significant changes in the AMR profiles of NTS.

## Supplementary Data

Supplementary material 1

Supplementary material 2

Supplementary material 3
