# Supplementary figures and images for: Genomic diversity of antimicrobial resistance in non-typhoidal Salmonella in Victoria, Australia
Source: Microb Genom. 2021 Dec 15;7(12):000725. doi: 10.1099/mgen.0.000725 (PMC8767345; doi:10.1099/mgen.0.000725)

A)

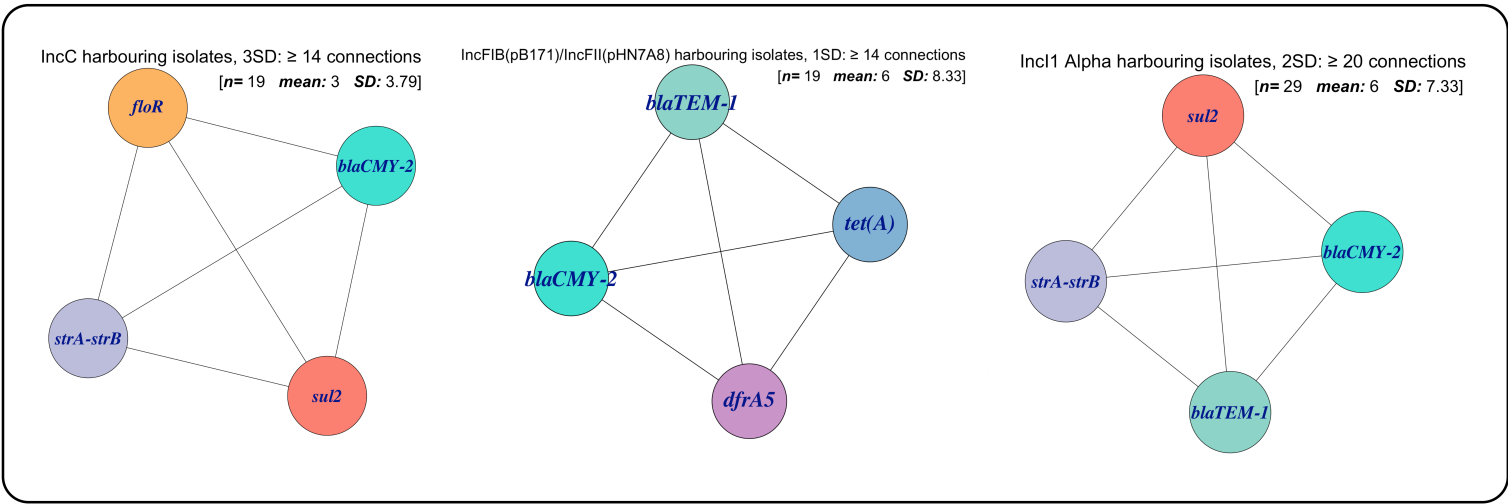

B)

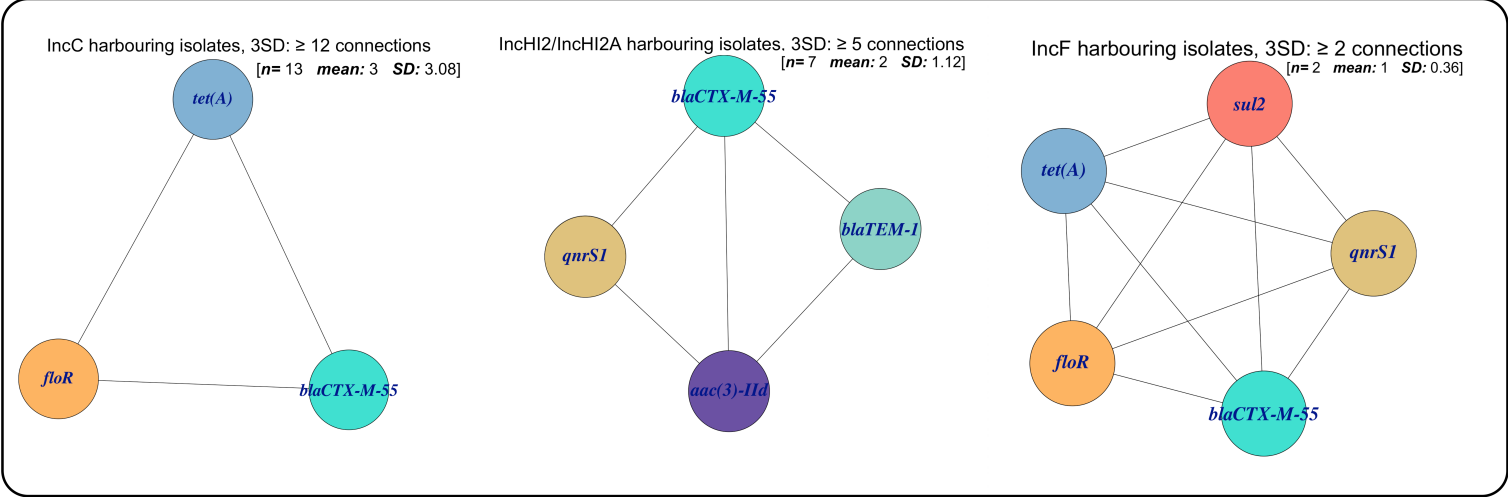

C)

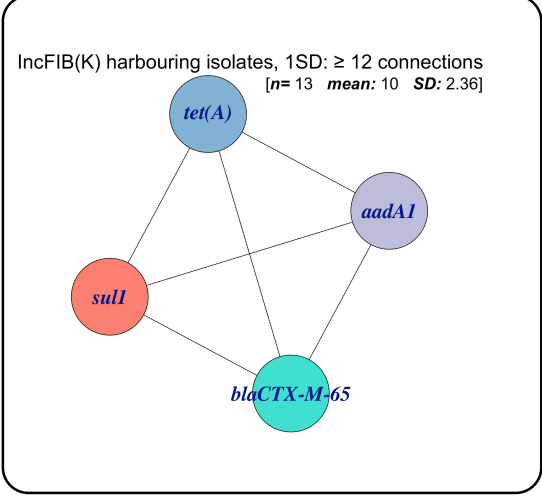

Supplement: Supplementary material 1 [file mgen-7-0725-s001.pdf]
