# Supplementary material for: Genomic diversity of antimicrobial resistance in non-typhoidal Salmonella in Victoria, Australia
Source: Microb Genom. 2021 Dec 15;7(12):000725. doi: 10.1099/mgen.0.000725 (PMC8767345; doi:10.1099/mgen.0.000725)
Supplement: Supplementary material 2 [file mgen-7-0725-s002.pdf]

Supplementary Figure 1. Co-occurring MDR patterns of *bla*$_{CMY-2}$, *bla*$_{CTX-M-55}$ and *bla*$_{CTX-M-65}$ plasmid types

The nodes represent genes, and the connecting branches (edges) represent the frequency of co-occurrence at a given threshold. The threshold of each co-occurring network is represented by the highest standard deviation (SD) that demonstrates MDR. The number of connections represent the number of isolates demonstrating that co-occurring pattern. A) Most common co-occurring AMR determinants demonstrating MDR in association with *bla*$_{CMY-2}$ plasmid types. B) Most common co-occurring AMR determinants demonstrating MDR in association with *bla*$_{CTX-M-55}$ plasmid types. C) Most common co-occurring AMR determinants demonstrating MDR in association with *bla*$_{CTX-M-65}$ plasmid types.

Supplementary Tables

Supplementary Table S1: Phenotypic and genotypic susceptibility profiles of all isolates in the study.
Supplementary Table S2: Summary table of final comparison between phenotypic and genotypic data with corresponding CLSI breakpoints for antimicrobials used in study.
Supplementary Table S3: Discordant isolates and their corresponding genotypic-phenotypic susceptibility results.
Supplementary Table S4: The prevalence rate of each resistance gene detected in concordant isolates.
Supplementary Table S5: Frequency of resistant and intermediate AMR determinant combinations according to antimicrobial class with its corresponding phenotypic susceptibilities.
Supplementary Table S6: Genomic profiles of multidrug resistant isolates harbouring antimicrobial resistant (AMR) determinants *bla*$_{CMY-2}$, *bla*$_{CTX-M-55}$ and *bla*$_{CTX-M-65}$.
